# Prevalence and pattern of basal skull fracture in head injury patients in an academic hospital

**DOI:** 10.4102/sajr.v23i1.1677

**Published:** 2019-03-13

**Authors:** Ntjeke S. Mokolane, Cornelia Minne, Alireza Dehnavi

**Affiliations:** 1 Department of Radiology, Sefako Makgatho Health Sciences University, Ga-Rankuwa, Pretoria, South Africa; 2 Department of Radiology, Dr George Mukhari Academic Hospital, Sefako Makgatho Health Sciences University, Ga-Rankuwa, Pretoria, South Africa; 3 Clinical Unit, Dr George Mukhari Academic Hospital, Ga-Rankuwa, Pretoria, South Africa

## Abstract

**Background:**

Basal skull fractures (BSFs) have been reported to be a major cause of morbidity and mortality in the literature, particularly in young male patients. However, there are limited data available on the aetiology, prevalence and patterns of such observed in South Africa.

**Objectives:**

To evaluate the prevalence and pattern of BSF in head injury patients referred to Dr George Mukhari Academic Hospital, Gauteng, South Africa.

**Methods:**

Patients of all ages with head injuries were considered for the study, and those who met the inclusion criteria were scanned using a 128-slice multidetector helical computed tomography (CT) machine after obtaining consent. Data were prospectively obtained over a 6-month period, interpreted on an advanced workstation by two readers and statistically analysed.

**Results:**

The prevalence of BSF in this study was found to be 15.2%. The majority of patients (80.5%) were under 40 years old, with a male to female ratio of 3:1. The most common aetiology of BSF was assault, which accounted for 46% of cases. The middle cranial fossa was the most frequently fractured compartment, while the petrous bone was the most commonly fractured bone. There was a statistically significant association between head injury severity and BSF, and between the number of fracture lines and associated signs of BSF (*p* < 0.001). The sensitivity of clinical signs in predicting BSF was 31%, while specificity was 89.3% (*p* = 0.004).

**Conclusion:**

The prevalence and pattern of BSF found were consistent with data from previously published studies, although, dissimilarly, assault was found to be the most common aetiology in this study.

## Introduction

According to Ryan et al.,^1^ the human skull consists of three components: calvarium, facial bones and mandible. The calvarium is further divided into the skull vault and the skull base, the latter of which consists of the paired orbital plates with the cribriform plate in the middle, the sphenoid bone with its lesser wings anteriorly and greater wings posteriorly, parts of the squamous temporal bone and petrous temporal bone and, lastly, the occipital bone posteriorly.^1^ The inner surface of the skull base is divided into anterior, middle and posterior cranial fossae by the sphenoidal ridge anteriorly and the petrous ridge posteriorly.^2^

A basal skull fracture (BSF) is defined as any fracture involving the floor of the anterior, middle or posterior cranial fossa that results either because of a direct local or remote force mechanism.^3^ Although most BSFs are thought to be minor in nature, if missed or diagnosed late, they could lead to debilitating complications such as neurovascular injuries, cerebrospinal fluid (CSF) leaks, meningitis or even death, especially if severe and/or associated with intracranial haemorrhage.^4–10^ In fact, head injuries have not only been reported to be a major public health problem but are also a leading cause of morbidity and mortality in younger patients (i.e. those under the age of 45 years) in the developed world.^3,5,8^ In most studies, the underlying mechanism of injury has been consistently reported to be road accidents, a phenomenon that can be attributed to various factors depending on geographic location.^3,6,11^

The reported prevalence of BSF varies greatly in the literature. In the developed world and Asian studies, BSF has been reported to have a prevalence of 3.5% – 24%, whereas studies conducted in Nigeria suggest higher figures of 33% – 46%.^4,7,12,13^ The main limitation of these findings in the present study is that they are derived from Western, Asian and Nigerian populations and therefore may not necessarily be generalisable to the local South African population. For South Africa, there is limited data in the literature regarding the prevalence and pattern of BSF. It is for this reason that we decided to evaluate the prevalence and pattern of BSF in the South African context.

The clinical presentation of patients with BSF varies and is determined by the severity of head injury and/or associated injuries elsewhere in the body. Some of the known clinical signs of BSF include racoon eye, rhinorrhoea, rhinorrhagia, anosmia, visual impairment, otorrhoea, otorrhagia, hearing loss, neurovascular injuries, Battle sign, phonation problems, vocal cord paralysis and/or aspiration.^4,6,14^ It is important to note that if a CSF leak secondary to BSF is missed, it may lead to meningitis and, ultimately, death.^14–17^ Identification of these clinical signs in the setting of trauma should always prompt the treating clinician to arrange for an urgent computed tomography (CT) scan of the brain or make a referral to a centre with CT scan facilities for early diagnosis and management so that complications can be avoided. This is supported by the fact that the literature has largely shown the presence of clinical signs to be a statistically significant predictor of BSF.^4,12^

## Materials and methods

A cross-sectional prospective and observational study was performed at Dr George Mukhari Academic Hospital (DGMAH) located in the Northwest district of Tshwane, Gauteng province, South Africa. DGMAH is a teaching hospital affiliated with the Sefako Makgatho Health Sciences University. The study included any patient with a head injury referred to the Radiology Department for a CT brain scan, including paediatric patients. Conversely, the exclusion criteria included previous head injury, recent brain surgery, pre-existing bone pathology, incomplete images and/or severe artefacts that could render the images unreadable.

A total of 210 patients who met the inclusion criteria were analysed over a period of 6 months, from June to November 2016. Patients were scanned with a Philips Ingenuity Core 128-slice multidetector helical CT scan machine (Phillips, Amsterdam, the Netherlands) using a built-in head trauma protocol with 1-mm slices and a bone kernel. Images were then analysed on an IntelliSpace Portal V6.0 Philips workstation (Phillips) that offers multiplanar reconstruction and three-dimensional (3D) capabilities. Images were interpreted by the lead researcher (a radiology registrar in the third year of training at the time of the study) and a qualified general radiologist with 6 years of experience, independently of one another.

BSF was diagnosed when there was a direct visualisation of a fracture line of the skull base on CT. In patients with signs such as brain contusion, intracranial haemorrhage, pneumocephalus, haemosinus or other features of intracranial or basal skull injury without direct visualisation of the fracture line, the term ‘associated signs’ was utilised for the purpose of our study. We did not however make a distinction between patients who had associated signs from skull base injury and those with skull vault injury as this was not the primary focus of the study.

Inter-reader variability was evaluated using Kappa statistics. The data were captured on an Excel spreadsheet (Microsoft Corp., Redmond, WA, USA) and analysed using the SAS (SAS Institute, Cary, NC, USA) and Statistical Package for the Social Sciences version 25 (IBM Corp., Armonk, NY, USA) software programs.

## Ethical consideration

The present study received ethical clearance from the research ethics committee of the Sefako Makgatho Health Sciences University prior to commencement (ethics approval no. SMUREC/M/10/2016: PG). Consent was obtained from all the participants after the details of the study were explained to them. The patients were anonymised to protect their identity and their details were kept safe and confidential, with no divulgement to a third party under any circumstances.

## Results

**FIGURE 1:**
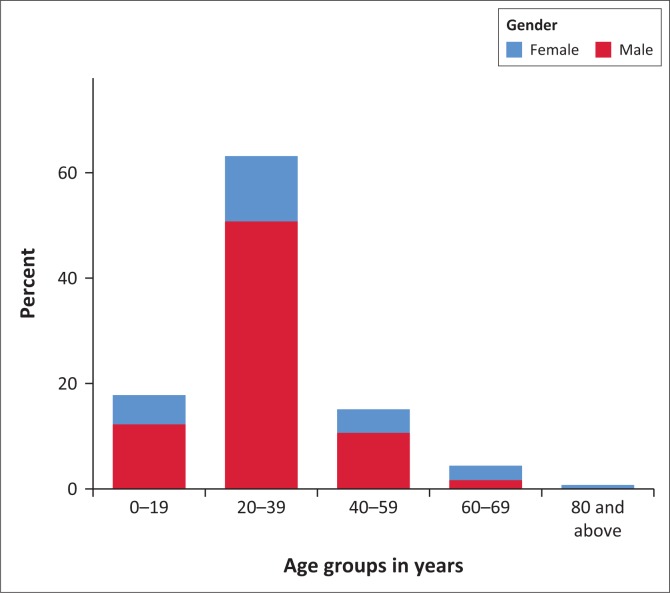
Age distribution of head injury patients (*n* = 200, missing data = 10).

The study analysed a total of 210 participants. There were 159 male and 51 female patients, with a male to female ratio of 3:1. Patients’ ages ranged from 1 to 81 years, with a mean of 29.61 years (standard deviation: 14.65 years) and a median (interquartile range) of 28 years (23–36 years). There were 10 patients whose ages could not be established; therefore, these individuals were not included in the age analysis. Figure 1 shows that the majority of the patients (*n* = 161; 80.5%) were under the age of 40 years.

Of the 210 patients analysed, 32 (15.2%) had BSF confirmed on CT scan, while 178 (84.2%) had no BSF. Of the 32 patients with BSF, 18 patients had middle cranial fossa (MCF) fractures, 15 patients had anterior cranial fossa (ACF) fractures and four patients had posterior cranial fossa (PCF) fractures. Five of these patients had fractures involving a combination of cranial fossa compartments. Three patients demonstrated combined ACF and MCF fractures, whereas two patients had combined MCF and PCF fractures. There was no ACF and PCF fracture combination nor was there an involvement of all three compartments in any of the patients.

**FIGURE 2:**
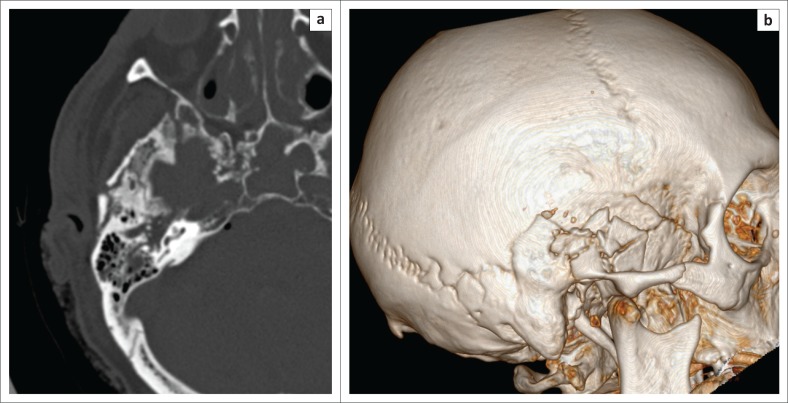
(a) Axial and (b) three-dimensional image of a complex petrous temporal bone fracture in combination with a comminuted, squamous temporal bone fracture.

**FIGURE 3:**
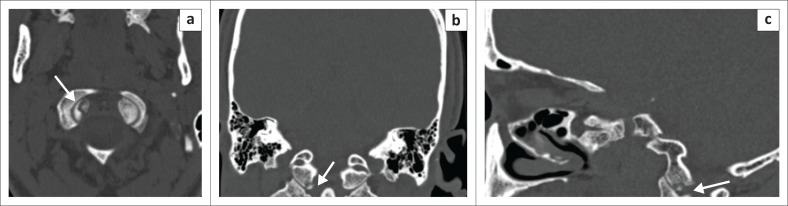
(a) Axial, (b) coronal and (c) sagittal computed tomography bone window with a right occipital condylar fracture (arrows).

Data in Table 1 reveal that the frontal sinus was the most fractured bone in the ACF, which was observed in seven patients. The same frequency of fractures was observed for a combination of different bones of the ACF. The MCF was the most frequently fractured compartment, with a total of 18 fractures recorded, as shown in Table 2. The most common isolated fractured bone without clinical signs in the MCF was squamous temporal, a finding which was observed on three occasions. Of the 18 fractures of the MCF, 12 were a combination of different bones of which nine did not show any clinical signs of BSF. The petrous bone fractures occurred in isolation in three patients and were longitudinal in all three individuals. Nine patients had a petrous bone fracture that occurred in combination with fractures of the other bones of the MCF, with an example illustrated in Figure 2. Table 3 shows the distribution of the types of petrous bone fractures. There was no violation of the otic capsule observed in any of the petrous bone fractures. The squamous occipital bone was the most frequently fractured bone in the PCF, occurring on three occasions, and none of the four patients with PCF fractures demonstrated clinical signs. Figure 3 shows occipital condylar fracture as an example of a posterior fossa fracture with intact skull base lines.

**TABLE 1:** Comparison of anterior cranial fossa clinical signs and fractures on computed tomography (*n* = 210).

Anterior fossa signs	Type of anterior cranial fossa fracture				
	No fracture	Frontal sinus fracture	Orbital roof fracture	Any combination	Total
No clinical signs	177	6	-	4	187
Rhinorrhagia	7	1	-	1	9
Raccoon eye	10	-	1	2	13
Any combination	1	-	-	-	1

**Total**	**195**	**7**	**1**	**7**	**210**

**TABLE 2:** Comparison of middle cranial fossa clinical signs and fractures on computed tomography (*n* = 210).

Middle fossa signs	Type of middle cranial fossa fracture					
	No fracture	Squamous temporal bone facture	Mastoid bone fracture	Petrous bone fracture	Any combination	Total
No signs	189	3	-	1	9	202
Otorrhoea	2	-	-	-	1	3
Otorrhagia	-	-	-	2	1	3
Haemotympanum	-	-	-	-	1	1
Any combination	1	-	-	-	-	1

**Total**	**192**	**3**	**-**	**3**	**12**	**210**

**TABLE 3:** Summary of petrous bone fractures on computed tomography

Nature of petrous bone involvement	Type of petrous bone fracture			Total
	Transverse	Longitudinal	Complex	
Isolated petrous bone fracture	-	3	-	3
Petrous bone fracture in combination with other bones	4	3	2	9

**Total**	**4**	**6**	**2**	**12**

There was a total of 63 (30%) patients who demonstrated associated signs of BSF on CT brain scan, of whom 28 had a BSF on CT, while the remaining 35 did not have a BSF on CT. This implies that 87.5% (28/32) of patients who had a BSF also had associated signs as revealed by CT, whereas 80.3% (143/178) who did not have BSF also had no associated signs (*p* < 0.001, chi-squared test). The following associated signs were observed in an order of decreasing frequency: haemorrhagic brain contusion (33), subarachnoid haemorrhage (28), brain oedema (26), blood in the paranasal sinuses (24), blood in the mastoid air cells (21), pneumocranium (19), blood in the middle ears (14), epidural hematoma (12), subdural hematoma (10), air in the temporomandibular joint (5) and intracranial foreign body (3).

Table 4 demonstrates that the majority of patients with BSF had a single fracture line, accounting for 56.3% (18/32) of the cases, followed by complex fractures of more than three fracture lines, as seen in 34% (11/32) of cases. It further shows that there was a statistically significant association between an increasing number of fracture lines and the presence of associated signs on CT scan, with an adjusted odds ratio (OR) of 3.89 (*p* < 0.001) and a corresponding 95% confidence interval (CI) of 1.93–7.88.

**TABLE 4:** Relationship between number of fracture lines and associated signs on computed tomography.

Associated signs	Number of fracture lines on CT					Total
	None	One	Two	Three	More than three	
Absent	143	2	-	-	2	147
Present	35	16	2	1	9	63

**Total**	**178**	**18**	**2**	**1**	**11**	**210**

Note: *p* < 0.001, simple binary logistic regression.

CT, computed tomography.

Table 5 shows that assault was the most common aetiology of head injury in this study, at 46.7% (98/210), followed by motor vehicle accidents (MVAs) and pedestrian vehicle accidents (PVAs) at 22% (47/210) and 15% (32/210), respectively. The proportions of patients who had BSF because of assault, fall, MVA, and PVA were all similar at approximately 15%, despite their respective absolute values being different. Patients with gunshot injuries, motor bike accidents (MBAs), other penetrating injuries and unknown aetiology as a mechanism of their injury had limited data; therefore, their percentages were not evaluated. Simple binary logistic regression with an adjusted OR of 3.26 (*p* < 0.001) and a corresponding 95% CI of 1.99–5.35 demonstrated that there was a significant association between head injury severity as measured by means of Glasgow Coma Scale (GCS) and the presence of BSF on CT scan. Table 5 further shows that 75% (158/210) of the patients with head injury had mild head injury with no BSF.

**TABLE 5:** Summary of severity of head injury and basal skull fracture in relation to mechanism of injury (*n* = 210).

Mechanism of injury	Basal skull fracture on CT														Subtotal		Grand total	
	No fracture: Head injury severity								Fracture present: Head injury severity									
	Mild		Moderate		Severe		Subtotal		Mild		Moderate		Severe					
	*n*	%	*n*	%	*n*	%	*n*	%	*n*	%	*n*	%	*n*	%	*n*	%	*n*	%
Assault	73	75	2	2	8	8	83	84.7	8	8	6	6	1	1	15	15.3	98	46
Fall	23	85	-	-	-	-	23	85.2	4	15	-	-	-	-	4	14.8	27	13
PVA	22	69	4	13	1	3	27	84.4	-		3	9	2	6	5	15.6	32	15
MVA	36	77	2	4	2	4	40	85.2	2	4	1	2	4	9	7	14.8	47	22
Gunshot	1	-	-	-	-	-	-	-	-	-	-	-	1	-	-	-	2	-
MBA	1	-	-	-	-	-	-	-	-	-	-	-	-	-	-	-	1	-
Stab	1	-	-	-	-	-	-	-	-	-	-	-	-	-	-	-	1	-
Unknown	1	-	-	-	1	-	-	-	-	-	-	-	-	-	-	-	2	-

**Total**	**158**	**-**	**8**	**-**	**12**	**-**	**-**	**-**	**14**	**-**	**10**	**-**	**8**	**-**	**-**	**-**	**210**	**-**

Note: Severity of head injury was measured by means of GCS, where mild is 13–15, moderate is 9–12 and severe is 3–8. *p* < 0.001, simple binary logistic regression.

MOI, mechanism of injury; CT, computed tomography; GCS, Glasgow Coma Scale; PVA, pedestrian vehicle accident; MBA, motor bike accident; MVA, motor vehicle accident.

Only 10 of 32 (31.3%) patients with BSF were positively predicted by means of clinical signs, while 159 of 178 (89.3%) patients with no BSF were identified in the absence of clinical signs (*p* = 0.004, Fisher’s exact test). This implies that, in our study, clinical signs had a sensitivity of 31.3%, a specificity of 89.3%, a positive predictive value of 34.4% and a negative predictive value of 87.8% for BSF as compared with CT brain scan. This study notably showed that the most common single clinical sign that ACF patients presented with was a racoon eye, which was observed in 13 patients, followed by rhinorrhagia in nine patients (Table 1). Conversely, the most common clinical signs of MCF were otorrhoea and otorrhagia, each sign presenting in three patients (Table 2). There were no clinical signs observed for PCF fractures.

As this study employed two readers, the inter-rater reliability was evaluated and there was 85% agreement between the two readers, with a *p*-value of < 0.001 and a 95% CI of 82.0–88.2. This finding was associated with a Kappa coefficient of 0.4655, which has a moderate weighting according to Altman.^18^

## Discussion

This study revealed that the majority of patients who presented with a head injury were young (i.e. under the age of 40 years) and predominantly men, with a male to female ratio of 3:1. Notably, these cohort characteristics are consistent with those in previous studies.^4,6,8^ A striking contrast between these studies and ours is that while the former have consistently shown vehicle-related accidents to be the most common aetiology of traumatic head injuries, our study found assault to be the highest cause, accounting for 46% (98/210) of all cases of traumatic head injury evaluated. This finding implies that there were high levels of interpersonal violence in this study population, despite a survey by Statistics South Africa reporting a moderate decline in assault cases between 2013 and 2017.^19^

The prevalence of BSF in this study was found to be 15.2% (32/210), irrespective of aetiology. This figure falls well within the 3.5% – 24% range that has been reported in studies from the United Kingdom, India and Hong Kong,^7,12,20^ whereas it is at least half of what has been published before in the Nigerian literature.^4,5,11^ The MCF was the most common fractured skull base compartment in this study followed by the ACF, with the PCF recording the least number of fractures. This distribution is consistent with those seen in previous studies. The predominance of MCF fractures is thought to be because of the presence of neurovascular foramina (which are presumed to be points of structural weakness) and thin bones in the MCF, which render it susceptible to fractures in comparison with the anterior and posterior compartments.^4,6^ Furthermore, the site of impact in the case of head injury tends to be from the sides or front.^2^

Within the middle cranial fossa, the most common fractured bone was the petrous bone. A total of six patients demonstrated a longitudinal fracture, while four had a transverse fracture and two had a complex fracture according to the traditional radiographic classification system based on fracture orientation.^21^ It should be noted, however, that this classification system has been criticised in the literature for its inability to predict clinical sequelae. The preferred newer classification system is otic capsule violating versus sparing of the otic capsule because of its clinical relavance.^9,17,21^ It was also found that 75% (9/12) of patients with petrous bone fractures also had fractures of other bones of the skull base. This suggests that, in a patient with a petrous bone fracture after a head injury, the radiologist interpreting the CT brain scan should actively look for fractures of other bones of the skull base.

It is essential that bone window with thin slices of 1 mm–1.15 mm should be used in the evaluation of basal skull fractures in order to minimise the chances of false negative outcomes in pursuit of these fractures. On the other hand, false positive outcomes because of misinterpretation of suture lines as fractures can be avoided by prior knowledge of the sites of suture lines, the cortical lining of the suture lines and a width of less than 2 mm in the suture line.^20^ In the presence of BSF, it is always important to assess the integrity of the base of skull lines as their disruption indicates cranio-cervical junction dissociation that is life-threatening.^22^

Of the five patients who had BSF involving combinations of skull base compartments, three had a mild head injury and two had a severe head injury as determined based on GCS with variable aetiologies. This suggests that BSF involving a combination of skull base compartments might have no direct relationship with the severity of head injury.

There was a statistically significant association between BSF and associated signs, with 87.5% (28/32) of patients with BSF having associated signs on CT scan (*p* < 0.001, chi-squared test). Thus, in patients who have associated signs on CT scan following a head injury, a fracture line should be sought so that the patient can receive the appropriate intervention(s). Thirty-five of 63 patients with associated signs on CT did not have BSF. This rate can be attributed to the wider inclusion of associated signs, such as contusions and subarachnoid haemorrhages. It was also demonstrated that 56% (18/32) of the included patients with BSF had a single fracture line, while 34.4% (11/32) had more than three fracture lines. A statistically significant association between the number of fracture lines and associated signs on CT scan meant that patients with more fracture lines were more likely to show associated signs on CT scans (adjusted OR: 3.89, 95% CI: 1.93–7.88; *p* < 0.001). It is therefore very important to note that, if a patient has multiple fracture lines on a CT brain scan following a head injury, associated signs should be sought and managed accordingly in order to prevent potential complications.

Clinical signs showed a low sensitivity of 31.3% (10/32) in predicting BSF in this study population. This is in contrast to the results of previous studies that have shown good clinical signs with sensitivity of 71% – 77% in predicting BSF.^4,12^ The low yield present in the current study can be explained by the fact that this study was not designed to evaluate the sensitivity of clinical signs in predicting BSF and the experience of the attending emergency room medical officers was not established. Conner et al.^7^ similarly reported a lower figure of 47%; however, it should be noted that evaluating the sensitivity of clinical signs was not the primary objective of their study. Having said that, this study showed a statistically significant association between head injury severity by means of GCS and BSF on CT scan, with an adjusted OR of 3.26 (*p* < 0.001) and a corresponding 95% CI of 1.99–5.35. This is even more applicable in our South African setting with limited resources, where some of the district hospitals do not have CT scanners on their premises and have to refer patients to other centres for imaging and further management. For medical officers in these hospitals, it means that patients with a low GCS, following head injury, should be referred urgently for CT brain scan in order to prevent or manage complications appropriately. Given that the majority of the patients in our study had mild head injury with no BSF, it is advisable that clinicians in our setting should familiarise themselves with the Canadian CT head rules in order to avoid unnecessary requests or referrals for head CT. This will aid in reducing the load on an already overburdened healthcare system without compromising the quality of care provided for head injury patients.^23^

Some of the limitations of our study include the fact that it was a purely epidemiological study that was performed over a short period of time and as such, the study sample might not be a true reflection of the South African population given its relatively small size and restricted geographic location. In addition, our study did not take the inclusion and exclusion criteria of the Canadian CT head injury rules into consideration.

## Conclusion

The findings of the study showed a prevalence of BSF of 15.2%, which is consistent with other percentages reported in the developed world and Asian studies. Our predominance of young male patients, mild head injuries, middle cranial fossa fractures and frequent involvement of the petrous bone are all findings consistent with those of previous studies. Contrary to previous studies, however, assault was the most common aetiology in the present study, reflecting the violent nature of our society. This finding appeals to law enforcement agencies to better ensure the safety and security of our citizens at all times.

The strong association between clinical assessment of head injury severity and BSF on CT scan implies that, in the absence of CT, patients’ referral to well-resourced centres should be expedited. In addition, radiologists should be vigilant regarding associated signs, such as intracranial bleeding in the presence of multiple fracture lines, when interpreting CT brain scans of head injury patients. It is recommended that similar studies should be conducted with much larger sample sizes and at other geographic locations in South Africa in order to obtain a much better reflection of the prevalence, pattern and aetiology of BSF in the country.

## References

[1] Ryan S, McNicholas M, Eustace S Anatomy for diagnostic imaging. 3rd ed. Edinburgh: Saunders Elsevier; 2011.

[2] Bell DJ, Mudgal P Base of the skull. Radiopaedia [serial online]. [cited 2018 Dec 30]. Available from: https://radiopaedia.org/articles/base-of-the-skull

[3] McElhaney JH, Hopper RH Jr., Nightingale RW, Myers BS Mechanisms of basilar skull fracture. J Neurotrauma. 1995;12(4):669–678. 10.1089/neu.1995.12.669 8683618

[4] Olabinri EO, Ogbole GI, Adeyele AO, Dairo DM, Malomo AO, Ogunseyinde AO Comparative analysis of clinical and computed tomography features of basal skull fractures in head injury in southwestern Nigeria. J Neurosci Rural Pract. 2015;6(2):139–144. 10.4103/0976-3147.153215 25883468 PMC4387799

[5] Emejulu JKC, Malomo O Head trauma in a newly established centre in Nigeria. East Cent Afr J Surg. 2008;13(1):86–94.

[6] Sivanandapanicker J, Nagar M, Kutty R, et al Analysis and clinical importance of skull base fractures in adult patients with traumatic brain injury. J Neurosci Rural Pract. 2018;9(3):370–375. 30069094 10.4103/jnrp.jnrp_38_18 PMC6050782

[7] Connor SEJ, Flis C The contribution of high-resolution multiplanar reformats of the skull base to the detection of skull-base fractures. Clin Radiol. 2005;60:878–885. 10.1016/j.crad.2005.04.003 16039923

[8] Agrawal A, Agrawal CS, Kumar A, Lewis O, Malla G, Chalise P Head injury at a tertiary referral centre in the eastern region of Nepal. East Cent AfriJ Surg. 2009;14(1):57–63.

[9] Jennett B Epidemiology of head injury. J Neurol Neurosurg Psychiatry. 1996;60:362–369. 10.1136/jnnp.60.4.362 8774396 PMC1073884

[10] Eisenberg HM, Howard E, Garry E Jr., et al Initial CT findings in 753 patients with severe head injury: A report from NIH Traumatic Coma Bank. J Neuorosurg. 1990;73:688–698. 10.3171/jns.1990.73.5.0688 2213158

[11] Adeyele AO, Olayemi O Basilar skull fracture: Outcome of acute care without antibiotic prophylaxis in a Nigerian neurosurgical unit. Turk Neurosurg. 2010;20(4):430–436. 10.5137/1019-5149.JTN.3038-10.1 20963690

[12] Goh KYC, Ahuja A, Walkden SB, Poon WS Is routine computed tomographic (CT) necessary in suspected basal skull fractures? Injury. 1997;28(5–6):353–357. 9764231 10.1016/s0020-1383(97)00024-7

[13] Johnson F, Semaan MT, Megerian CA Temporal bone fracture: Evaluation and management in the modern era. Otolaryngol Clin North Am. 2008;41:597–618. 10.1016/j.otc.2008.01.006 18436001

[14] Collins JM, Krishnamoorthy AK, Kubal WS, Johnson MH, Poon CS Multidetector CT of temporal bone fractures. Semin Ultrasound CT MR. 2012;33:418–431. 10.1053/j.sult.2012.06.006 22964408

[15] Bell RB, Dierks EJ, Homer L, Potter BE Management of cerebrospinal fluid leak associated with craniomaxillofacial trauma. J Maxillofac Surg. 2004;62:676–684. 10.1016/j.joms.2003.08.032 15170277

[16] Gerbino G, Roccia F, Benech A, Caldarelli C Analysis of 158 frontal sinus fractures: Current surgical management and complications. J Cranio-Maxillofac Surg. 2000;28:133–139. 10.1054/jcms.2000.0134 10964548

[17] Ash GJ, Peter J, Bass DH Antimicrobial prophylaxis for fractured base of skull in children. Brain Inj. 1992;6(6):521–527. 10.3109/02699059209008149 1393186

[18] Altman DG Practical statistics for medical research [homepage on the Internet]. 1st ed. London: Chapman and Hall; 1990 Available from: https://www.crcpress.com/Practical-Statistics-for-Medical-Research/Altman/p/book/9780412276309/

[19] Statistics South Africa Victims of crime survey [Stats SA] [homepage on the Internet]. [updated 2017 Sep 28; cited 2018 Oct 29]. Available from: https://www.statssa.gov.za

[20] Wani AA, Ramzan AU, Raina T, et al Skull base fractures: An institutional experience with review of literature. Indian J Neurotrauma. 2013;10:120–126. 10.1016/j.ijnt.2013.05.009

[21] Little SC, Kesser BW Radiographic classification of temporal bone fractures clinical predictability using a new system. Arch Otolaryngol Head Neck Surg. 2006;132(12):1300–1304. 10.1001/archotol.132.12.1300 17178939

[22] Hacking C Base of skull lines. Radiopaedia [serial online]. [cited 2018 Dec 30]. Available from: https://radiopaedia.org/cases/base-of-skull-lines-annotated-images?lang=us

[23] Canadian CT head injury/trauma rule [MDCALC] [homepage on the Internet]. [cited 2018 Dec 30]. Available from: https://www.mdcalc.com/canadian-ct-head-injury-trauma-rule

